# The first troglobitic species of freshwater flatworm of the suborder Continenticola (Platyhelminthes) from South America

**DOI:** 10.3897/zookeys.470.8728

**Published:** 2015-01-12

**Authors:** Stella Teles de Souza, Ana Laura Nunes Morais, Lívia Medeiros Cordeiro, Ana Maria Leal-Zanchet

**Affiliations:** 1 Instituto de Pesquisas de Planárias and Programa de Pós-Graduação em Biologia, Universidade do Vale do Rio dos Sinos – UNISINOS, 93022-000 São Leopoldo, RS, Brazil; 2 Instituto de Biociências da Universidade de São Paulo, Departamento de Zoologia, Rua do Matão, trav. 14, nº 321, Cidade Universitária,CEP: 05508-090, São Paulo, SP, Brazil

**Keywords:** New cave-dwelling species, subterranean diversity, Brazilian savannah, planarians, triclads

## Abstract

Brazilian cave diversity, especially of invertebrates, is poorly known. The Bodoquena Plateau, which is located in the Cerrado Biome in central Brazil, has approximately 200 recorded caves with a rich system of subterranean water resources and high troglobitic diversity. Herein we describe a new troglobitic species of *Girardia* that represents the first obligate cave-dwelling species of the suborder Continenticola in South America. Specimens of the new species, which occur in a limestone cave in the Bodoquena Plateau, in the Cerrado biome, are unpigmented and eyeless. Species recognition in the genus *Girardia* is difficult, due to their great morphological resemblance. However, the new species can be easily recognized by a unique feature in its copulatory apparatus, namely a large, branched bulbar cavity with multiple diverticula.

## Introduction

Despite a significant development of the speleobiology in Brazil over the last two decades, species diversity of Brazilian cave fauna has been highly underestimated (Ferreira 2005, Trajano and Bichuette 2010). More than 10,000 caves have been documented in Brazil, but this may represent only 10% of the total number of Brazilian caves, especially considering the extensive karst regions and other potential areas in the country (Auler et al. 2001, Galvão and Cruz 2012). There is much heterogeneity in the degree of knowledge about different karst areas and associated taxa. More troglobitic species have been described in well-known areas from southeast Brazil, such as Alto Ribeira, São Paulo, than in central and north Brazil (Trajano 2000, Trajano and Bichuette 2010, Cordeiro et al. 2014).

The Bodoquena Plateau, in central Brazil (Mato Grosso do Sul), has approximately 200 recorded caves with a rich system of subterranean water resources from the phreatic level (Sallun et al. 2010, Neto 2010). The region has high troglobitic diversity, especially in freshwater ecosystems (Trajano et al. 2000, Trajano and Bichuette 2010; Cordeiro et al. 2014). A total of 34 species of obligate cave-dwelling animals has been recorded from the Bodoquena Plateau, including catfishes and many invertebrates, such as amphipods, spelaeogriphaceans and oligochaetes (Godoy 1986, Gnaspini and Trajano 1994; Gnaspini et al. 1994, Pinto da Rocha 1995, Moracchioli 2002; Costa-Junior 2004, Pires-Vanin 2012, Cordeiro et al. 2013, Cordeiro et. al. 2014). Among the invertebrates, a triclad species hereby assigned to the suborder Continenticola was found in one of the caves.

The diversity of freshwater triclads of the suborder Continenticola in the Neotropical region is considered to be low, and most of the species belong to the Dugesiidae genus *Girardia* Ball (Sluys et al. 2005). However, extensive areas of South America remain unexplored, such as the Cerrado Biome in central Brazil. The genus *Girardia* ranges from South to North America and contains 46 species (Sluys 2005, Tyler et al. 2006-2013). According to several authors, species recognition is difficult in this genus, due to their great morphological resemblance (Hyman 1939, Du Bois-Reymond Marcus 1953, Sluys 1996, Sluys et al. 1997, 2005). Most species are recognized on the basis of a combination of morphological characters rather than unique features (Sluys 1996, Sluys et al. 1997).

Triclad diversity in South American subterranean habitats is largely unknown. Kawakatsu and Froehlich (1992) and Trajano and Bichuette (2010) recorded unidentified Dugesiidae species in caves from three different locations, one of which is herein described as new. In addition, Kawakatsu and Froehlich (1992) documented the presence of troglophilous specimens of the suborder Continenticola in three Brazilian caves in Pará State, northern Brazil that they assigned to *Girardia paramensis* (Fuhrmann). Recently, the first troglobitic triclad of the suborder Cavernicola in South America was recorded in a limestone cave in northeastern Brazil (Leal-Zanchet et al. 2014). Herein we describe a new species of freshwater triclad, the first troglobitic representative of the suborder Continenticola in South America that can be recognized by a unique feature of its copulatory apparatus.

## Material and methods

**Figures 1–3.**
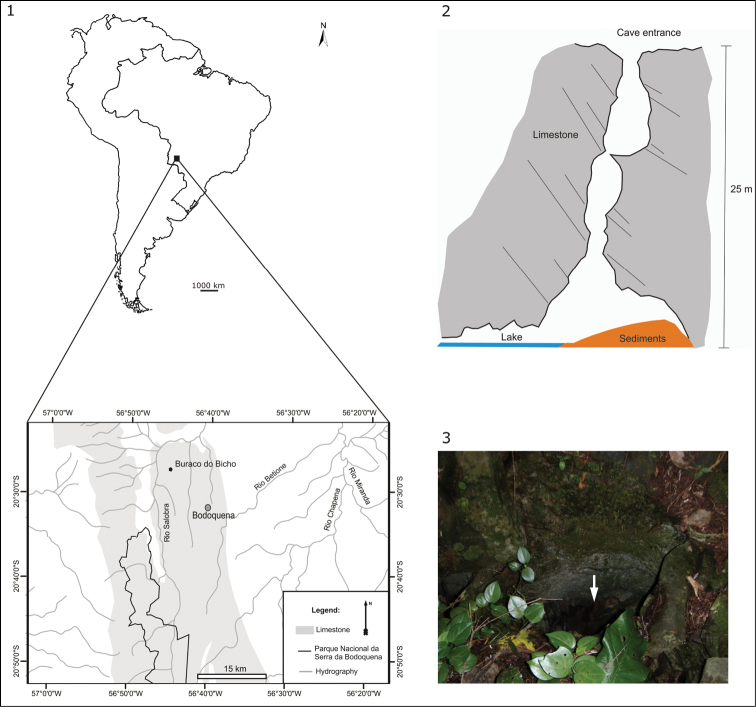
Type-locality of *Girardia multidiverticulata*: **1** location of the “Buraco do Bicho” cave, in Bodoquena Plateau, Mato Grosso do Sul, Brazil, showing the range of limestone outcrops and the adjacent “Serra da Bodoquena” National Park **2** schematical drawing of the “Buraco do Bicho” cave from where the flatworms were sampled **3** cave entrance (arrow).

Specimens were collected from the limestone cave “Buraco do Bicho”, located at 266 m a.s.l. in the karst area of Bodoquena Plateau (20°33’50”S and 56°43’50”W), Mato Grosso do Sul, Brazil (Fig. 1). The type-locality is situated in the southern part of the Cerrado biome. The Brazilian savanna is dominated by a tropical climate with a dry winter (type Aw of Köppen’s classification), but the southern portion has a tropical humid climate with warm winter (type Cfa of Köppen’s classification). The mean annual rainfall is approximately 1,400 mm year^–1^, and the mean annual temperature is about 22 °C to 24 °C (Sallun et al. 2010).

The flatworms were directly sampled from a lake (10 m^2^) in the cave, at a depth of 25 m from the narrow entrance of the cave (Figs 2–3). The lake has a maximum depth of 1.60 m and shows clear waters over a clayey bottom with the parent rock exposed in some places.

**Figures 4–6.**
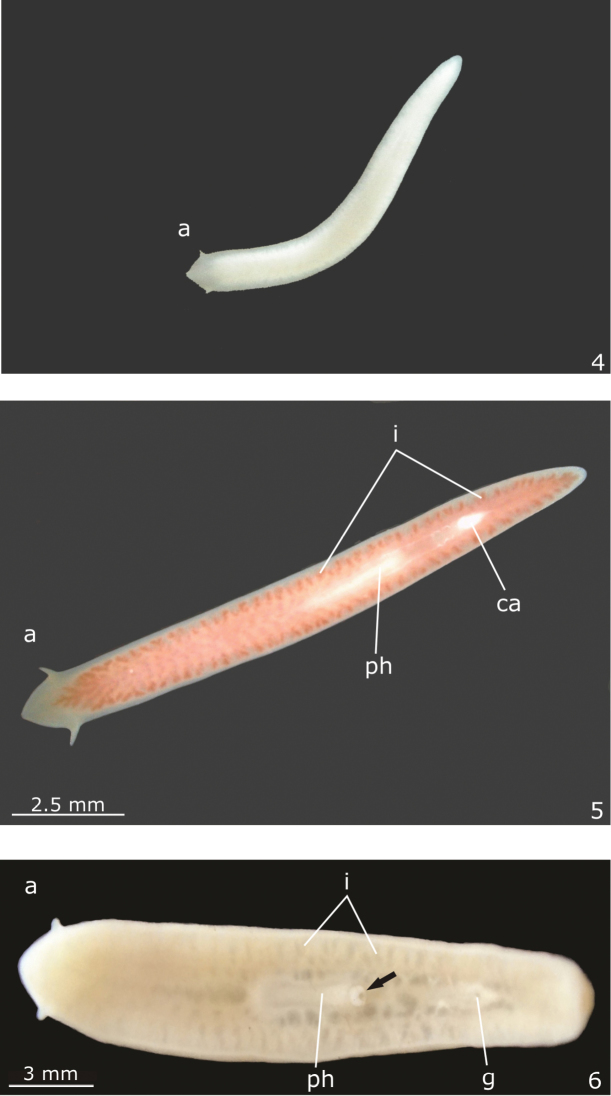
*Girardia multidiverticulata*: **4** photograph of a live specimen in ventral view soon after sampling **5** photograph of a live specimen, in ventral view, fed at the laboratory **6** photograph of a preserved specimen in ventral view. The tip of the pharynx is protruded (arrow) through the mouth. Scale bar for the Fig. 4 not available.

Live specimens were photographed in the field and in the laboratory (Figs 4–6). Specimens were analysed under a stereomicroscope and fixed with 10% Formalin. They were dehydrated and embedded in Paraplast. This material was sectioned at 5−7 µm and stained with hematoxyline/eosine or Goldner’s Masson (Romeis 1989). Colour descriptors, based on the uptake of dyes of particular colours, were used for classifying secretions with trichrome methods.

Type-material was deposited in the following reference collections: Museu de Zoologia da Universidade do Vale do Rio dos Sinos, São Leopoldo, Rio Grande do Sul, Brazil (MZU), and the Helminthological Collection of Museu de Zoologia da Universidade de São Paulo, São Paulo, São Paulo State, Brazil (MZUSP).

The flatworms were maintained in a permanently dark laboratory under a temperature of 24 °C for three years. They were kept in small tanks and fed weekly with live *Artemia salina*.

### Abbreviations used in the figures

a: anterior tip; bc: bulbar cavity; br: brain; ca: copulatory apparatus; cb: copulatory bursa; cbc: bursal canal; cg: cyanophil glands; ceg: cement glands; cf: circular muscle fibers; ci: cilia; cm: circular cutaneous musculature; cs: cyanophil secretion; de: dorsal epidermis; di: diverticula of the bulbar cavity; eg: erythrophil glands; ej: ejaculatory duct; em: external pharyngeal musculature; es: esophagus; fa: female atrium; g: gonopore; go: gonoduct; i: intestine; im: internal pharyngeal musculature; in: insunk nuclei; lf: longitudinal muscle fibers; lm: longitudinal cutaneous musculature; lu: pharyngeal lumen; m: mouth; ma: male atrium; mm: muscles; n: nerve cord; o: ovary; om: oblique cutaneous musculature; ov: oviducts; pb: penis bulb; pg: penis glands; ph: pharynx; phg: pharyngeal glands; php: pharyngeal pouch; pp: penis papilla; r: rhabdites; rg: rhabditogen glands; s: sperm; sd: sperm duct; sg: shell glands; sv: spermiducal vesicle; t: testes; ve: ventral epidermis; vi: vitellaria; xg: xanthophil glands.

## Systematic acount

### Order Tricladida Lang, 1884 Suborder Continenticola Carranza et al., 1998 Family Dugesiidae Ball, 1974 Genus *Girardia* Ball, 1974

#### Girardia multidiverticulata sp. n.

Taxon classificationAnimaliaTricladidaDugesiidae

http://zoobank.org/147CB963-DECB-4125-985D-124B306B5EA0

##### Material examined.

*Holotype*. MZUSP PL.1573: “Buraco do Bicho” cave, Bodoquena Plateau, Mato Grosso do Sul (MS), Brazil, July 2011, *coll*. L. M. Cordeiro & R. Borghezan, sagittal sections on 18 slides.

*Paratypes*. “Buraco do Bicho” cave, Bodoquena Plateau, MS, Brazil, July 2011, *coll*. L. M. Cordeiro & R. Borghezan. MZU PL.00184: sagittal sections on 61 slides; MZU PL.00185: sagittal sections on 8 slides; MZU PL.00186: transverse sections on 16 slides.

##### Etymology.

The species name refers to the multiple diverticula of the bulbar cavity.

##### Diagnosis.

Blind and unpigmented *Girardia* species characterized by a branched bulbar cavity with multiple irregular diverticula.

##### Description.

Live specimens unpigmented and eyeless (Figs 4–6). Head highly triangular with long and pointed auricles, which become moderately sized and almost rounded after fixation (Fig. 6); posterior tip rounded (Figs 4–6). Preserved specimens up to 20 mm long and 3 mm wide (Table 1). Mouth and gonopore located at the posterior half of the body (Table 1, Fig. 6).

**Table 1.** Measurements, in mm, of specimens of *Girardia multidiverticulata*, sp. n. DG: distance of gonopore from anterior end; DM: distance of mouth from anterior end. The numbers given in parentheses represent the position relative to body length. * Measurements after fixation; ** Measurements after histological processing; -: not measured.

	Holotype MZUSP PL.1573	Paratype MZU PL.00184	Paratype MZU PL.00185	Paratype MZU PL.00186
Length*	16	20	12	14
Length**	12.5	15	9	12
Width*	2	3	2	3
DM	9 (72%)*	9 (60%)**	6 (67%)**	-
DG	11 (88%)*	10.5 (70%)**	7 (78%)**	-

**Figures 7–10.**
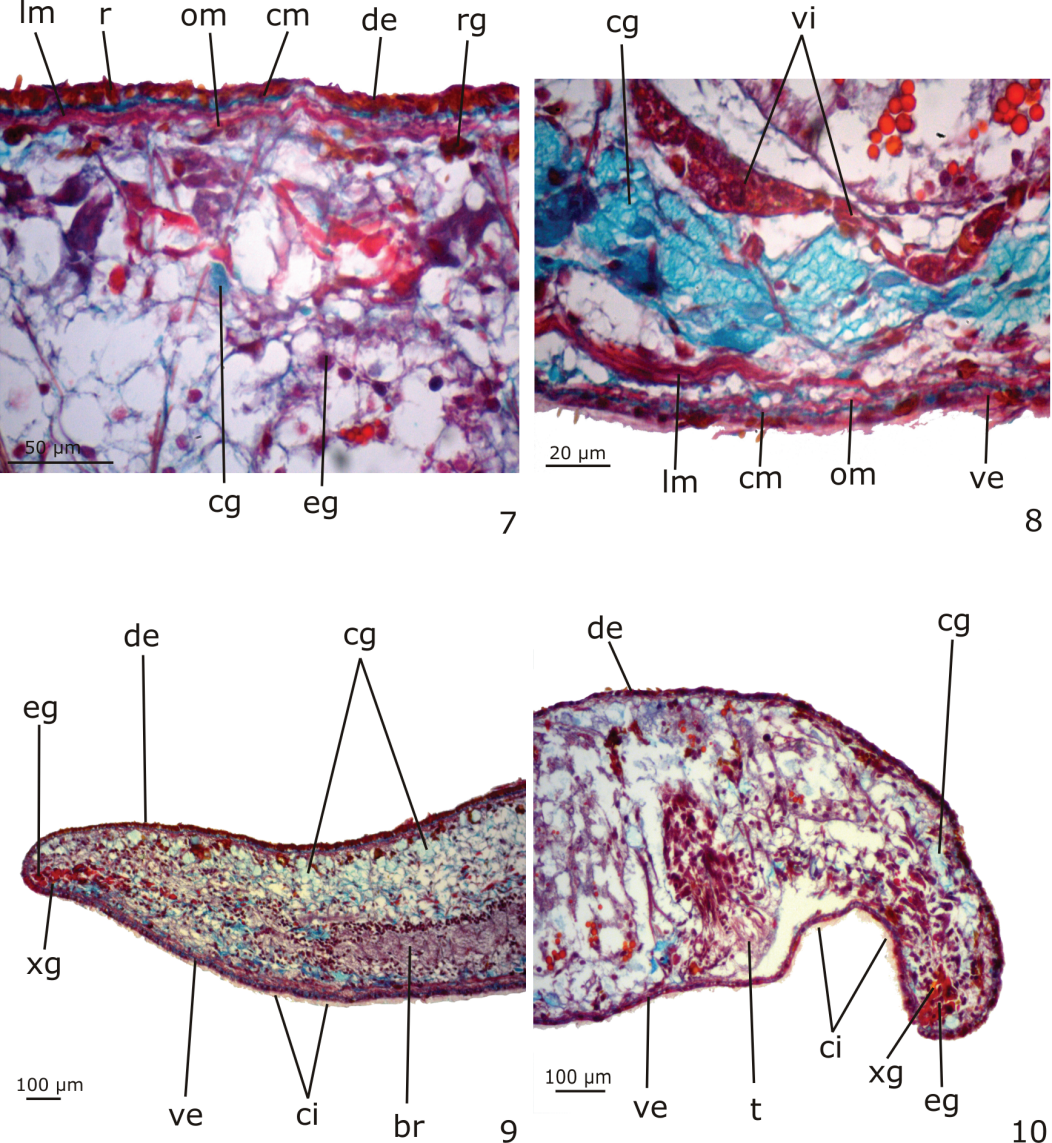
*Girardia multidiverticulata*, holotype in sagittal section: **7–8** dorsal and ventral surfaces of the body, respectively **9–10** anterior and posterior tips of the body, respectively.

*Epidermis* (Figs 7–10). Columnar epithelium, ciliated on the ventral body surface (Figs 7, 10). The whole epidermis receives secretions of three types of glands: (1) xanthophil, rhabidtogen secretion (rhammites); (2) erythrophil, fine granular secretion; (3) cyanophil amorphous secretion (Figs 7–10). Rhammites are more densely distributed at the dorsal surface (Fig. 7). The erythrophil glands and a fourth type of gland, with xanthophil, granular secretion, concentrate their openings medially at the anterior and posterior tips of the body (Figs 9–10) as well as at the body margins. Cyanophil glands become numerous towards the anterior tip (Fig. 9).

*Cutaneous musculature* (Figs 7–8). Three layers, viz. a thin subepithelial circular layer, followed by an oblique layer with decussate fibers and a thicker layer of longitudinal muscle. Dorsal and ventral cutaneous musculatures show similar height in the pre-pharyngeal region (10–13 µm thick in the holotype).

*Sensory organs*. The auricular sensory organs are lined with densely ciliated, low cuboidal epithelium, with insunk nuclei. Few secretory cells open through this epithelium. The cutaneous musculature is very thin at the level of the sensory organs.

**Figures 11–13.**
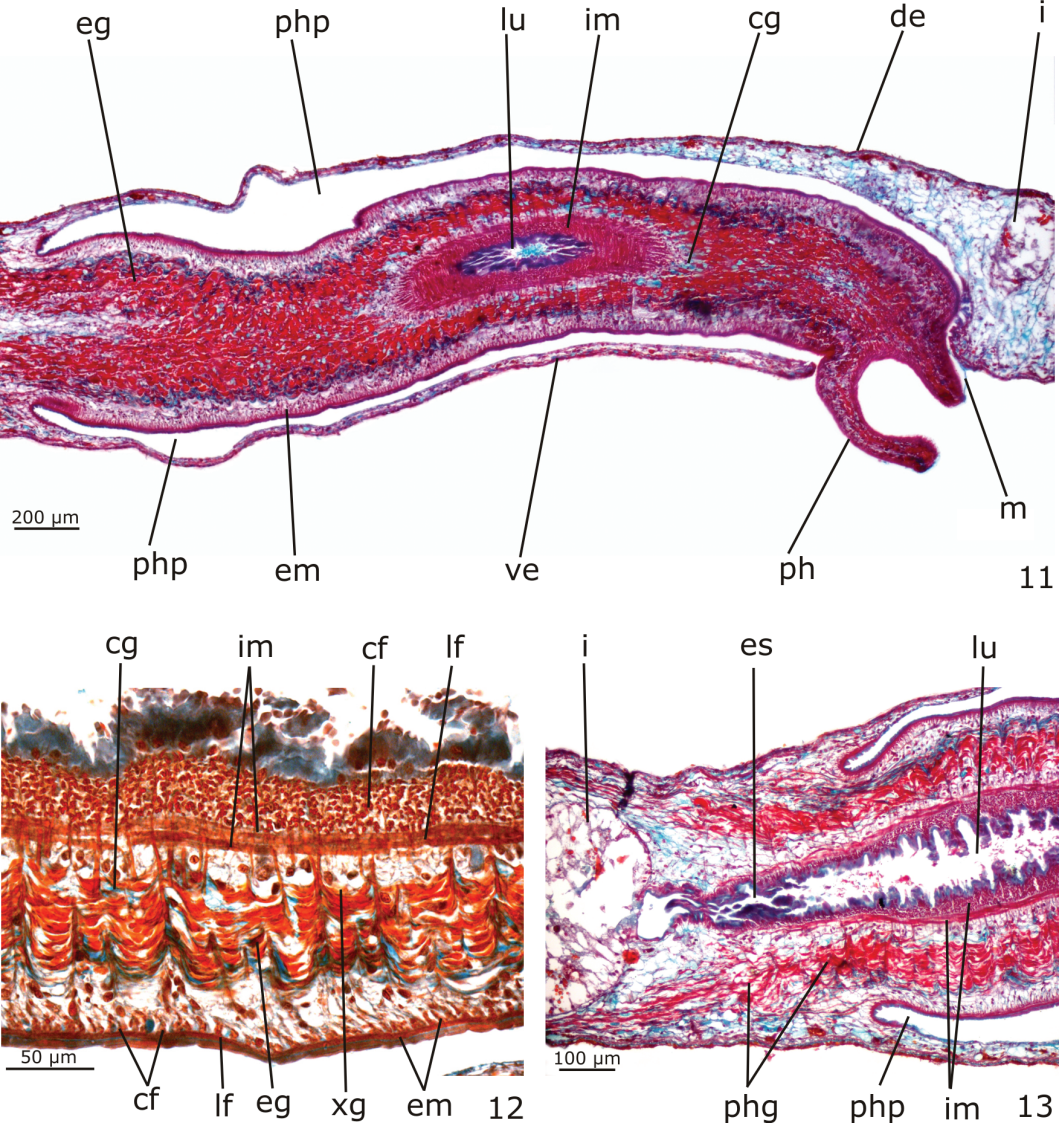
*Girardia multidiverticulata*, holotype in sagittal section: **11** pharynx in general view **12** detail of pharyngeal musculature and glands **13** detail of the esophagus.

*Digestive system* (Figs 5–6, 11–13). Pharynx cylindrical, non-pigmented; between about 1/4th and 1/6th of the body length. It is located in the posterior half or in the median third of the body (Figs 5–6). Mouth at the posterior end of the pharyngeal pouch (Fig. 11). Pharynx lined by cuboidal ciliated epithelium with insunk nuclei; pharyngeal lumen lined by non-ciliated, columnar epithelium with some insunk nuclei. Pharyngeal glands of the usual three types (xanthophil, cyanophil and erythrophil glands). Outer musculature of the pharynx constituted of a thin subepithelial layer of longitudinal muscle, followed by a thin layer of circular muscle, each about 4 µm thick in the holotype. Inner pharyngeal musculature composed of a thick subepithelial layer of circular muscle (30–60 µm thick in the holotype), followed by a layer of longitudinal muscle (15–20 µm thick in the holotype) (Figs 11–13). An esophagus, about 1/6 of the pharyngeal length, connects the pharynx with the intestine (Fig. 13). The esophagus is lined by a flat to cuboidal epithelium with insunk nuclei; it is coated with a thin muscularis containing circular fibers near the intestine, gradually becoming thicker towards the pharynx and similar to the inner pharyngeal musculature. Intestine with the usual tricladid form (Fig. 5), with the anterior intestinal trunk extending onto the posterior part of the brain.

*Male reproductive system* (Figs 10, 14–19, 24–26).

**Figure 14.**
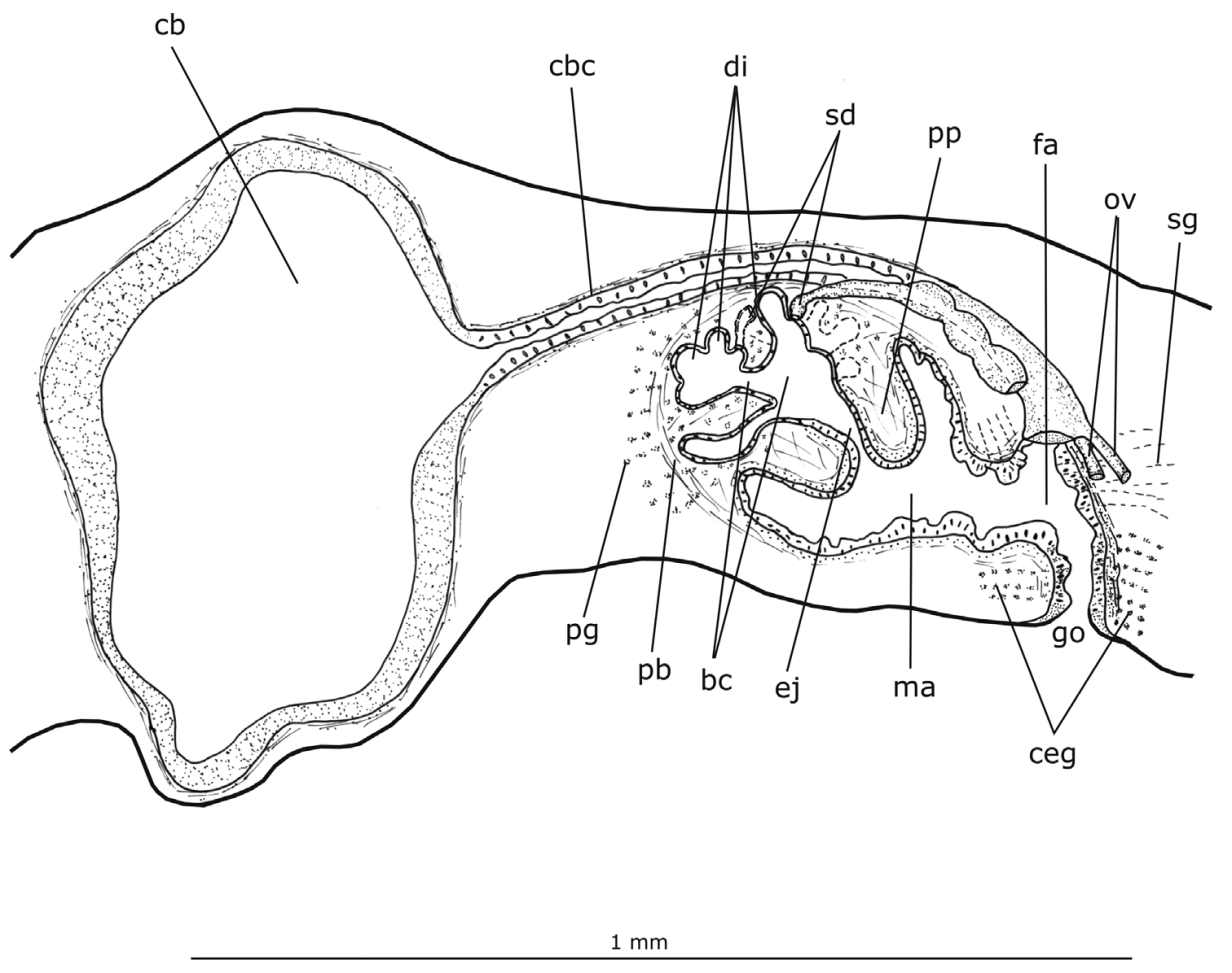
*Girardia multidiverticulata*: sagittal composite reconstruction of the copulatory apparatus of the holotype.

**Figures 15–20.**
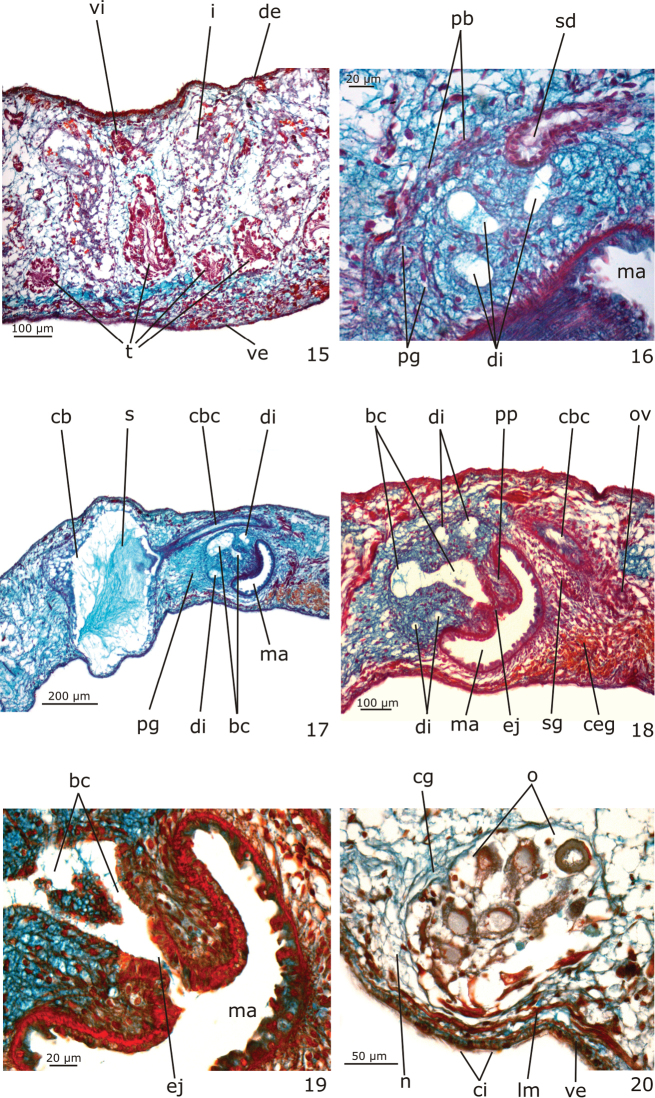
*Girardia multidiverticulata*, holotype in sagittal section: **15** testes in the anterior body region **16** detail of the opening of a sperm duct into a diverticulum of the bulbar cavity **17–18** copulatory apparatus in general view **19** detail of the male copulatory organs **20** ovary.

**Figures 21–26.**
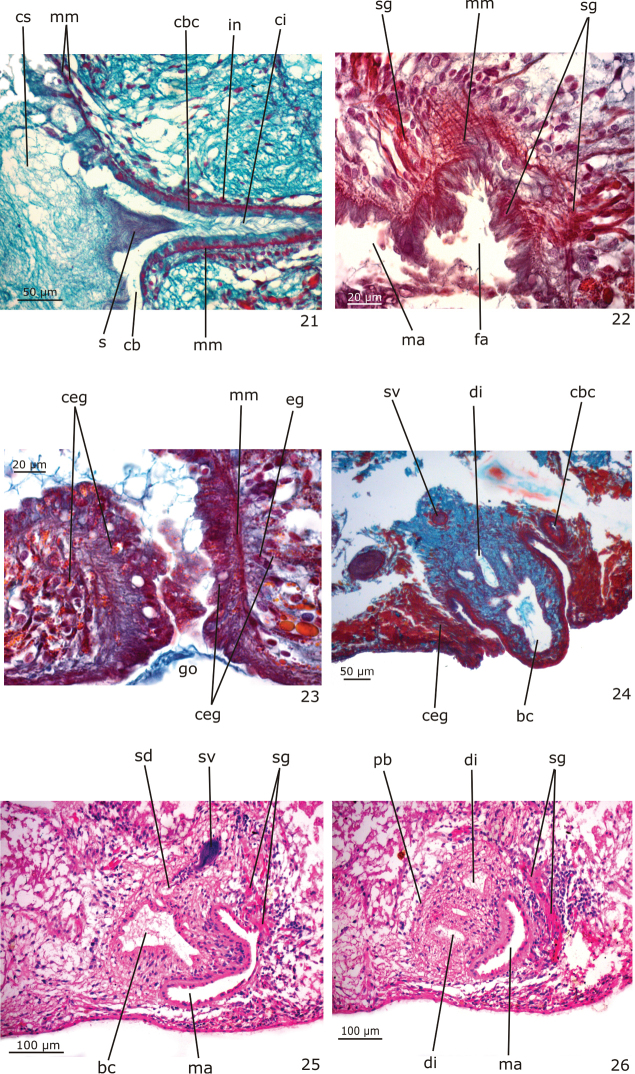
*Girardia multidiverticulata*, holotype in sagittal section (**21–23**); paratypes MZU PL.00186 in transverse section (**24**) and MZU PL.00184 in sagittal section (**25–26**): **21** detail of the copulatory bursa and its canal **22** detail of the proximal part of the female atrium **23** gonoduct **24** protruded penis papilla **25–26** male copulatory organs.

Numerous testicular follicles, 100–200 µm in diameter in the holotype, arranged in one irregular row on each side of the body. They are situated mainly ventrally (Fig. 15), but may occupy the whole body height; some are situated dorsally. Testes extend from about 2 mm from the anterior tip in the holotype (equal to 16% of body length), just behind the brain, to the posterior end of the body (Fig. 10). Sperm ducts form spermiducal vesicles laterally to the pharynx, diminishing in diameter close to their opening into the bulbar cavity (Fig. 16). Laterally to the copulatory apparatus, they ascend, forming a loop, and turn anteriad. Sperm ducts separately penetrate the penis bulb, and open laterally into the large, branched bulbar cavity which contains various irregular diverticula (Figs 14, 16, 18, 24–26). The short ejaculatory duct narrows towards its opening at the tip of the penis papilla. The latter is a stubby, symmetrical cone, obliquely oriented in the male atrium (Figs 14, 18–19, 25–26).

Sperm ducts lined with a ciliated, cuboidal epithelium, becoming flattened in the spermiducal vesicles; they are coated with a circular muscle layer (3 µm thick in the holotype). The large penis bulb consists of a loose connective tissue containing abundant gland necks of penis glands and interwoven muscle fibers (Figs 14, 16). Bulbar cavity lined with a non-ciliated, cuboidal to flat epithelium, underlain with a weak and inconspicuous muscle layer. Numerous penis glands with extrabulbar cell bodies and mixed secretion open into the bulbar cavity (Figs 14, 16–17). This secretion has a cyanophil external part and an erythrophil internal core. In addition, few erythrophil penis glands with extrabulbar cell bodies open into the bulbar cavity. Ejaculatory duct lined with non-ciliated, columnar epithelium, and surrounded by a thin muscularis (about 3 µm thick in the holotype) composed of a subepithelial layer of circular muscle and a layer of longitudinal muscle. Erythrophil glands have abundant openings into the distal, narrow portion of this duct (Fig. 19). Penis papilla covered with a non-ciliated, columnar epithelium that becomes flat towards the tip of the papilla. Muscularis of penis papilla (5–9 µm thick in the holotype) composed of a thick subepithelial layer of circular fibres and a thin subjacent layer of longitudinal fibres (Fig. 19). Few penis glands with amorphous, cyanophil secretion and with fine granular, erythrophil secretion open through the epithelium of the penis papilla. Cyanophil glands with extrabulbar cell bodies; erythrophil glands with intrapapillar cell bodies. Male atrium lined with a non-ciliated, cuboidal to columnar epithelium, the cells of which have an irregular height and cyanophil cytoplasm (Fig. 19). The male atrial muscularis (4–5 µm thick in the holotype) is constituted of a thick subepithelial layer of circular fibres, followed by a thin layer of longitudinal fibres. Glands with cyanophil amorphous secretion and erythrophil glands with fine granular secretion open into the male atrium. Cyanophil glands with extrabulbar cell bodies, and erythrophil glands with subepithelial cell bodies.

*Female reproductive system* (Figs 8, 14, 17, 20–23).

Vitellaria well developed (Fig. 8), located between intestinal branches. Ovaries ovoid (Fig. 20), 150–200 µm in diameter in the holotype. They are situated dorsally to the ventral nerve cords, at about the same transversal level as the anteriormost testes and in close proximity to the brain (about 0.9 mm behind it in the holotype). Ovovitelline ducts arising from the lateral surface of the ovaries and running backwards dorsally to the nerve cords. At about the level of the gonoduct, the ovovitelline ducts turn medially, and separately open into the most distal, postero-ventral part of the bursal canal, in close proximity to each other. Copulatory bursa large and ovoid (Figs 14, 17). Bursal canal long, curving towards the ventral surface of the body and opening into the short female atrium (Figs 14, 17). Gonoduct almost straight (Fig. 14, 23).

Ovovitelline ducts lined with ciliated, cuboidal epithelium with insunk nuclei and covered mainly by circular muscle fibres (2–3 µm thick in the holotype). Copulatory bursa lined with non-ciliated, columnar epithelium composed of cells with erythrophil secretion and cells with heavily stained, cyanophil secretion; it is covered by a thin muscle coat constituted by interwoven longitudinal and circular muscle fibres (5–8 µm thick in the holotype). The bursa of the holotype contains sperm and cyanophil secretion in its lumen (Figs 17, 21); some spermatozoids are absorbed by its epithelial cells. Bursal canal lined with a ciliated, cuboidal to columnar epithelium with cyanophil cytoplasm (Fig. 21). The muscularis of the bursal canal (3–4 µm thick in the holotype) is constituted of interwoven circular and longitudinal muscle fibres (Fig. 21). There are some insunk nuclei and cell bodies of xanthophil glands around the copulatory bursa and bursal canal. Female atrium lined with a ciliated, tall columnar epithelium, the cells of which show irregular height. The muscularis of the female atrium (6 µm thick in the holotype) is constituted of a subepithelial layer of circular fibres, followed by a layer of longitudinal fibres (Fig. 22). Numerous glands with fine granular, erythrophil secretion (shell glands) and few cyanophil glands open into the female atrium. Gonoduct lined by ciliated, tall columnar epithelium, and coated with a subepithelial layer of circular muscle, followed by a layer of longitudinal muscle (about 9 µm thick in the holotype) (Fig. 23). Abundant cement glands with coarse granular, xanthophil secretion (Fig. 23) and numerous glands with heavily stained, cyanophil amorphous secretion discharge into the gonoduct. Both cell types have long cell necks and their cell bodies are located in the mesenchyme. Few glands with fine, erythrophil secretion and subepithelial cell bodies also open into the gonoduct (Fig. 23).

##### Geographical distribution.

Known only from the type-locality (“Buraco do Bicho” cave), Bodoquena Plateau, Mato Grosso do Sul, Brazil.

##### Variability.

In paratype MZU PL.00186 with contracted body, the penis papilla protrudes into the gonoduct and the bulbar cavity formes two main proximal chambers and one large distal one (Fig. 24). The distal portion of the bursal canal and the female atrium of this paratype were elongated and protruded towards the ventral surface of the body (Fig. 24). Paratype MZU PL.00184 has a more elongate, conical and truncated penis papilla occupying almost the whole cavity of the male atrium (Fig. 25). Despite the fact that this specimen is mature, it has a small copulatory bursa with narrow cavity, probably due to a different physiological state in relation to the other specimens. In this paratype, stained with Hematoxyline/Eosine, the penis glands showed an amorphous, chromophobous secretion, shell glands were stained deep pink (Figs 25–26) and cement glands showed chromophobous, coarse granular secretion.

### Ecology

There was a density of about 5 to 10 flatworms per m^2^ in the lake that constitutes the type-locality of *Girardia multidiverticulata*. Other invertebrates, such as the spelaeogriphacean *Potiicoara brasiliensis* Pires, the amphipod *Megagidiella* sp. and an undetermined species of troglomorphic oligochaete, were also observed. The water level did not vary between June and August 2011, when field work was performed. The recorded values of temperature and conductivity were 23.1 °C and 0.55 mS.cm^-1^, respectively.

Flatworms maintained in the laboratory reproduced sexually and produced stalked egg capsules. Usually 2 to 3 specimens hatched from each egg capsule.

## Discussion

Due to the lack of eyes and body pigmentation, the troglobitic *Girardia multidiverticulata* differs from the majority of its congeners, which are pigmented, epigean organisms. It can be differentiated from the hipogean *Girardia mckenziei* (Mitchell & Kawakatsu) from Chiapas, Mexico, which has a smaller body length, dorsal surface with a slight, microscopic pigmentation and minute eyes (Mitchell and Kawakatsu 1973). The new species herein described is similar to two other troglobite dugesiids, *Girardia typhlomexicana* (Mitchell & Kawakatsu) and *Girardia barbarae* (Mitchell & Kawakatsu), from Tamaulipas, Mexico, which are blind and eyeless (Mitchell and Kawakatsu 1972). However, both have a small body length, up to 8 mm, whereas mature specimens of the new species are between 12 mm and 20 mm long after fixation. In addition, live specimens of *Girardia multidiverticulata* show long and pointed auricles in contrast to the moderate-sized auricles of *Girardia typhlomexicana* and *Girardia barbarae*. *Girardia multidiverticulata* also differs from the troglophilous species *Girardia guatemalensis* (Mitchell & Kawakatsu), from Tamaulipas, Mexico, which has a pigmented body with two small eyes (Mitchell and Kawakatsu 1972, Kawakatsu and Mitchell 1981), and from the troglophilous specimens of *Girardia paramensis*, with pigmented body and a pair of eyes, recorded in Pará State, northern Brazil by Kawakatsu and Froehlich (1992).

Regarding the reproductive system, *Girardia multidiverticulata* has large, mainly ventral testes that occupy most of the dorso-ventral space of the body height, a large, branched bulbar cavity with multiple diverticula, and a stubby penis papilla. This combination of characteristics cannot be found in other species of *Girardia* from epigean or hipogean environments. The epigean species *Girardia anderlani* (Kawakatsu & Hauser) from the vicinity of São Leopoldo, southern Brazil, also has a large bulbar cavity, but with only two main chambers. In addition, this species has mainly ventral testes in two or three longitudinal rows and a conical and asymmetrical penis papilla (Kawakatsu et al. 1983). *Girardia multidiverticulata* shares an intermingled muscle coat around the copulatory bursa with the epigean species *Girardia bursalacertosa* Sluys (Sluys et al. 2005), but this feature is more developed in the latter than in the new species. In addition, *Girardia bursalacertosa* has an almost tubular bulbar cavity and a small copulatory bursa (Sluys et al. 2005), among other distinctive features.

Concluding, in comparison to other species of *Girardia*, most of which with very similar reproductive systems, the troglobitic *Girardia multidiverticulata* shows a unique feature in its copulatory apparatus, namely a large and branched bulbar cavity with multiple diverticula. Additionally, the new species has a combination of other characteristics of its external and internal morphology that differentiate it from its congeners.

## Supplementary Material

XML Treatment for Girardia multidiverticulata
